# Prevalence of reproductive tract and sexually transmitted infections among symptomatic and asymptomatic women, validity of syndromic management, in urban and periurban low to mid socioeconomic neighbourhoods of North Delhi: an observational study

**DOI:** 10.1136/bmjph-2024-001791

**Published:** 2025-10-27

**Authors:** Neeta Dhabhai, Barsha Gadapani Pathak, Gitau Mburu, Deepak More, Ranadip Chowdhury, Teodora E C Wi, Leena Chatterjee, Devjani De, Rita Kabra, James Kiarie, Ndema Habib, Arjun Dang, Manvi Dang, Aradhna Bhargav, Binish Jawed, Sarmila Mazumder

**Affiliations:** 1 Society for Applied Studies, New Delhi, India; 2 Department of Sexual and Reproductive Health and Research, UNDP/UNFPA/UNICEF/WHO/World Bank Special Programme of Research, Development and Research Training in Human Reproduction, World Health Organization, Geneva, Switzerland; 3 Department of Global HIV Hepatitis and Sexually Transmitted Infections Programmes, World Health Organisation, Geneva, Switzerland; 4 Dr Dangs Lab LLP, New Delhi, India; 5 Department of Microbiology, Vardhman Medical College and Safdarjung Hospital, New Delhi, India

**Keywords:** Public Health, Sexually Transmitted Diseases, Sexual Health

## Abstract

**Introduction:**

Reproductive tract infections (RTIs) including sexually transmitted infections (STIs) result in major reproductive health morbidity worldwide. There is a paucity of recent data on laboratory-confirmed prevalence in India of the curable pathogens responsible, *Neisseria gonorrhoeae* (NG), *Chlamydia trachomatis* (CT), *Trichomonas vaginalis* (TV), *Bacterial vaginosis* (BV) and *Candida albican*s (CA), with a significant proportion being asymptomatic. This study aims to determine the diagnostic accuracy of the syndromic approach and the prevalence of symptomatic and asymptomatic infections.

**Method:**

An observational study was conducted with 440 married, reproductive age women in low-income and middle-income neighbourhoods of Delhi. Vaginal swabs were collected irrespective of symptoms. Nucleic acid amplification technique was used for NG, TV and CT, gram stain for CA and Nugent’s criteria for BV.

Statistical analysis was done using STATA V.16.1. Categorical variables were analysed using the binomial exact method. Sensitivity, specificity, positive and negative predictive value, and likelihood ratios were calculated for the syndromic approach. Logistic regression was performed to assess associated factors.

**Result:**

262 of 440 women had a positive laboratory test. BV was 37%, CA 12.7%, CT, NG and TV were <1%. 56% of asymptomatic women had a laboratory confirmed RTI. Sensitivity and specificity of syndromic approach were 62% and 45%. Every 1-year increase in age of women was associated with an 8% reduction in the odds of having a lab-confirmed STI/RTI (OR=0.92).

**Conclusions:**

BV and CA were most prevalent infections. A syndromic approach had low sensitivity and specificity; young age was a risk factor.

**Trial registration number:**

CTRI/2020/03/023954.

**WHAT IS ALREADY KNOWN ON THIS TOPIC**

**WHAT THIS STUDY ADDS**

- This study draws attention to the asymptomatic burden of RTI/STI; it also brings cognisance to the fact that we need to include gold standard molecular laboratory methods to improve the diagnostic efficiency of the syndromic approach of management. Vaginal dysbiosis is more prevalent than assumed and needs a targeted approach.

**HOW THIS STUDY MIGHT AFFECT RESEARCH, PRACTICE OR POLICY**

- The study will provide useful information to the national programme by understanding the prevalence of laboratory confirmed RTI/STIs as well as diagnostic validity of syndromic approach. As a policy measure, a targeted high-risk approach to RTI/STI would help to pick up asymptomatic women and assist them to access treatment. Addition of molecular diagnostic methods would help to prevent antimicrobial resistance. Research needs to be directed to develop high sensitivity and specificity point-of-care tests using molecular technology. With an existing large prevalence of *Bacterial vaginosis*, research also needs to be directed to its prevention.

## Introduction

 Reproductive tract infections and sexually transmitted infections (RTIs/STIs) represent a significant global public health threat, impacting sexual and reproductive health worldwide. They contribute to substantial morbidity, including infertility, chronic pelvic pain, cancers and pregnancy complications. Notably, the WHO estimates that over 1 million STIs occur daily, with the majority remaining asymptomatic.^1^ These ‘hidden epidemics’, as termed by the Institute of Medicine, present a major challenge.^2^

India faces a substantial burden of RTIs/STIs, with an estimated 30–35 million episodes occurring annually (6%).^3^ However, the true scale of these infections remains elusive due to a lack of widespread screening strategies. The concern is about undetected asymptomatic STIs as they fuel transmission, distort disease burden data and can indirectly lead to overtreatment. Asymptomatic individuals unknowingly spread infections, sustaining community transmission. For example, many chlamydia and gonorrhoea cases are asymptomatic yet infectious.^4^

Underdiagnosis underestimates prevalence, hindering public health planning and missed opportunities for treatment. Paradoxically, it can also cause overtreatment, as clinicians may empirically treat, based on symptoms or risk, leading to unnecessary antibiotic use and resistance. Addressing this requires better screening and point of care diagnostics for effective control and rational treatment.^5^

Furthermore, recent research unveils a potentially crucial link between vaginal dysbiosis (a condition characterised by imbalances in the vaginal microbiome), increasing susceptibility to bacterial STIs such as *Neisseria gonorrhoeae* (NG), *Chlamydia trachomatis* (CT) and *Trichomonas vaginalis* (TV), as well as viral ones like HIV and HPV.^6 7^ Bacterial vaginosis (BV), the most common form of vaginal dysbiosis, has been implicated in adverse pregnancy outcomes, including second-trimester miscarriage, preterm birth and postcaesarean section endometritis.^7^ Especially, multiple sexual partners and inconsistent condom use are closely associated with an increased risk of BV.^8^

Given the dynamic and geographically diverse nature of the STI/RTI infections, the WHO recommends periodic prevalence and aetiologic surveys to accurately estimate the burden and inform national management guidelines.^9^ This study, leveraging the WING trial, presented a unique and valuable opportunity to conduct a community-based study on the prevalence of infections and to evaluate the validity of syndromic management.

Historically, most prevalence studies have been conducted in hospital or clinical settings, where only symptomatic individuals are typically seen.^10^ The objectives of the study were to assess the current prevalence of the most common bacterial RTIs/STIs (CT, NG, TV, BV and *Candida albicans* (CA) using gold-standard laboratory tests, including molecular diagnostic technology, to evaluate the validity of syndromic management and to identify associated demographic, behavioural, sexual and clinical factors. By including asymptomatic women, the study aimed to close critical knowledge gaps and contribute to more effective, evidence-based STI/RTI control strategies.

## Methods

### Study design and population

This was an analytical cross-sectional study conducted among married women aged 18–30 years with no or one child, residing in low-income to lower-middle-income neighbourhoods of Delhi, India. The aim was to investigate the prevalence of RTIs/STIs in this specific demographic. Women were recruited after exiting the WING study. This individually randomised factorial trial assessed the impact of an integrated community-level package (health, nutrition, WASH and psychosocial care) delivered during preconception, pregnancy and the first 2 years of life. 13 500 married women aged 18–30 years, residing in lower and lower-middle socioeconomic neighbourhoods of Delhi, living with their husbands, having no or one child, and desiring more children, were enrolled and randomised to receive either the preconception intervention package or routine care until pregnant or for 18 months post-enrolment. On pregnancy confirmation, a second randomisation assigned women to additional interventions or routine care. This approach resulted in a 24% reduction in low birth weight and a 51% reduction in stunting at 24 months of age, compared with the controls.^10^

### Sample size

Assuming a 6% prevalence of STIs in Indian adults with a 32% laboratory confirmation rate, a sample size of 440 women was calculated for a 95% CI with 15% relative precision.^11^

### Study procedures

The study started in January 2021 and was completed in early December 2022. The study protocol with methodology has been published elsewhere.^12^ Women who exited from WINGS were contacted on phone by the study team after at least 14 days from their exit from the trial. This study was explained and their willingness to participate was ascertained. Those who agreed were invited to the study clinic to participate. Written individual informed consent was obtained before enrolment in the study clinic, for quantitative data collection, collection of endocervical swabs and for interviews by research assistants. Women who were unable to read, the consent form was read out by the worker and for those who were unable to sign, a thumb imprint was taken. An impartial literate witness witnessed (counter-signed) all such consent forms. The informed consent form was translated into simple Hindi language which was easily read and understood.

### Inclusion and exclusion criteria

All consenting women were eligible; they were further screened for timing of sampling and rescheduled if they reported menstruation, sexual intercourse, vaginal douching or insertion of vaginal tablets in the last 48 hours as blood, semen and medication could potentially interfere with test results. Women who declined to consent were excluded.

### Data collection

Structured questionnaires were used to collect data about sociodemographic variables and sexual behaviour. Clinical data was entered in a real-time electronic tracker by the study gynaecologists

### Clinical examination, sample collection and transportation

Detailed history followed by clinical examination was performed for all women irrespective of the presence of symptoms (vaginal discharge—nature and type—colour, odour and quantity, burning while passing urine, low backache, itching, pain in the lower abdomen, rash, ulcers, dyspareunia, groin swelling, dysmenorrhoea) of RTI/STI by study gynaecologists. Signs of RTI/STI were documented. Vaginal and endocervical swabs were collected in prelabelled specified containers (for gram stain and nucleic acid amplification technique (NAAT)), stored between 2°C–8°C and transported daily in cool boxes (2°C–8°C) to the testing laboratory.^12^

### Laboratory procedures

Tests were performed at a National Accreditation Board for Testing and Calibration Laboratories accredited laboratory. Nugent’s scoring criteria (gold standard with ~77% specificity, 90% sensitivity) was used for diagnosis of BV (score ≥7).^13^ CA was identified by yeast cells and pseudo hyphae in the vaginal smear.

NAAT, a real-time PCR having the highest specificity and sensitivity (98%–100%) among the available conventional tests, was used to detect CT, NG and TV.^14^ We used cartridge-based NAAT Gene Xpert platform which is an automated in vitro diagnostic test for qualitative detection and differentiation of DNA of CT, NG and TV and is performed on Cepheid GeneXpert Instrument.^15^ Reports generated were reviewed and delivered to participants within 72 hours.

### Quality control and quality assurance

Kit verification was done before study initiation, as CT/NG kits were FDA approved while TV kits were labelled for research use only. We used the NAT trol verification panel comprising 17 samples containing known CT, NG and TV of high, medium and low concentration provided by Zeptometrix. 16,18100% concordance was achieved, excluding one run failure due to cartridge error. Each sample had to pass the three inbuilt kit checks, sample processing control, sample adequacy control and probe check control.^19 20^ Of the 440 samples, two were repeated due to blood contamination. For gram stain, *Staphylococcus aureus* (ATCC25923) and *Escherichia coli* (ATCC25922) were used as controls for each slide. 10 samples of every 100 tests were sent to the Apex Regional STD Laboratory at the tertiary care government hospital for interlab comparison as per protocol, 39 out of 40 samples matched.

### Definition

#### Syndromic approach

The syndromic management approach is based on the identification of consistent groups of symptoms and easily recognised signs (syndromes), with the provision of treatment which covers the majority of, or the most serious, organisms responsible for producing the syndrome. The entry point to a syndromic approach is the presence of symptoms.

#### Management of RTI/STI

Women who were clinically diagnosed as RTI/STI were treated as per the National Guidelines, medication and condoms were provided free of charge.^21^ Additional treatment was provided based on the laboratory report. Asymptomatic women who needed treatment were called to the study clinic and medication was provided. Counselling for prevention of RTI/STI was done, with partner notification and provision of medication where needed. Referral for further treatment, where indicated, was to the collaborative tertiary hospital.

#### Statistical analysis and outcome measures

We used STATA V.16.1 (StataCorp) for statistical analyses. Categorical variables were analysed using the binomial exact method to calculate proportions and 95% CIs. For continuous variables, means (SDs) or medians (IQRs) were calculated. Sensitivity, specificity, positive (PPV) and negative predictive values (NPV), and positive and negative likelihood ratios were calculated for diagnosing infections using gold standard laboratory methods compared with the syndromic approach. (Detailed definitions are shown in online supplemental material).

Logistic regression was used to assess associations with laboratory-confirmed cases of RTI/STI with demographic, behavioural, sexual and clinical risk factors. Variables with a p<0.20 in univariable analysis were considered for inclusion in the initial multivariable logistic regression model, following standard recommendations to avoid omitting potentially important predictors at the screening stage.^22 23^ A backward stepwise selection process was then used, removing variables with p>0.05. Variables not significant in the initial univariable analysis or the initial multivariable model were subsequently reintroduced one by one to evaluate their effect on the final model. The model diagnostics were performed to assess the adequacy and fit of the final multivariable logistic regression model. This included checking for multicollinearity using variance inflation factors, assessing model discrimination with the area under the receiver operating characteristic curve and evaluating goodness-of-fit using the Hosmer-Lemeshow test.

## Results

440 eligible women participated in the study, their characteristics are described in table 1. More than 90% of the 440 women were married housewives. About 60% were aged between 25 and 30 years, 63% had >10 or more years of schooling, 31% were overweight or obese, 9% underweight and 50% stayed in joint families.

**Table 1:** Baseline characteristics of the women enrolled in the study

Characteristics of participants	Women (N=440); n (%)		Men/husbands* (N=440); n (%)	
Individual characteristics				
Age (in years)	Category	n (%)	Category	n (%)
	20–24	96 (21.82)	19–24	67 (15.23)
	25–28	198 (45)	25–30	267 (60.68)
	29–32	123 (27.95)	31–36	96 (21.82)
	33–36	23 (5.23)	37–43	10 (2.27)
Occupation	Working†	28 (6.36)	Private job	330 (75.00)
	Housewife	412 (93.64)	Other jobs‡	97 (22.04)
			No work	13 (2.95)
Education (in years)				
No formal education	23 (5.23)		11 (2.50)	
1–5	40 (9.09)		33 (7.50)	
6–9	101 (22.95)		87 (19.77)	
10–13	163 (37.05)		195 (44.32)	
>13	113 (25.68)		114 (25.91)	
Marital status				
Married	435 (98.86)		435 (98.86)	
Single	1 (0.23)		1 (0.23)	
Divorced	1 (0.23)		1 (0.23)	
Widowed	3 (0.68)		3 (0.68)	
BMI (kg/m^2^) (n=439)§				
<18.5 (kg/m^2^)	40 (9.11)		–	
18.5–24.99 (kg/m^2^)	263 (59.91)		–	
≥25.0 (kg/m^2^)	136 (30.98)		–	
Household characteristics				
Type of family living in the same household				
Nuclear	161 (36.59)			
Extended*	60 (13.64)			
Joint	219 (49.77)			
Wealth quintile				
Lowest and second	177 (40.23)			
Middle	87 (19.77)			
Fourth and highest	176 (40.00)			
Religion				
Hindu	367 (83.41)			
Muslim	68 (15.45)			
Others	5 (1.14)			
Caste				
Scheduled caste (SC)¶	151 (34.32)			
Scheduled tribe (ST)**	6 (1.36)			
Other backward class (OBC)††	111 (25.23)			
None of them	172 (39.09)			

All values are numbers (percentages) unless stated otherwise.

Primary education—5 years (consists of basic understanding of subjects like mathematics, science and languages).

Secondary education—7 years (this builds on primary education, introducing students to more specialised subjects and preparing them for higher education or vocational paths).

Post-secondary education—3–4 years (encompasses higher studies pursued after completing secondary education, including undergraduate, postgraduate and doctoral programmes).

* Extended family: An extended family encompasses the nuclear family along with other relatives such as grandparents, aunts, uncles and cousins, who may or may not live together but maintain close relationships; Joint family: A joint family, also known as a Hindu Undivided Family, includes multiple generations living under one roof, sharing resources and responsibilities.

† Working women=daily wage earner, self-employed and private job.

‡ Other job=Govt. Job, self-employed and daily wage earner.

§ BMI missing for 1 participant.

¶ SC: Historically disadvantaged groups recognised by the government of India for affirmative action/reservation.

** ST: Indigenous communities with distinct cultures, also recognised for affirmative action/reservation.

†† OBC: Socially and educationally disadvantaged groups identified by the government.

BMI, body mass index.

**Figure 1:**
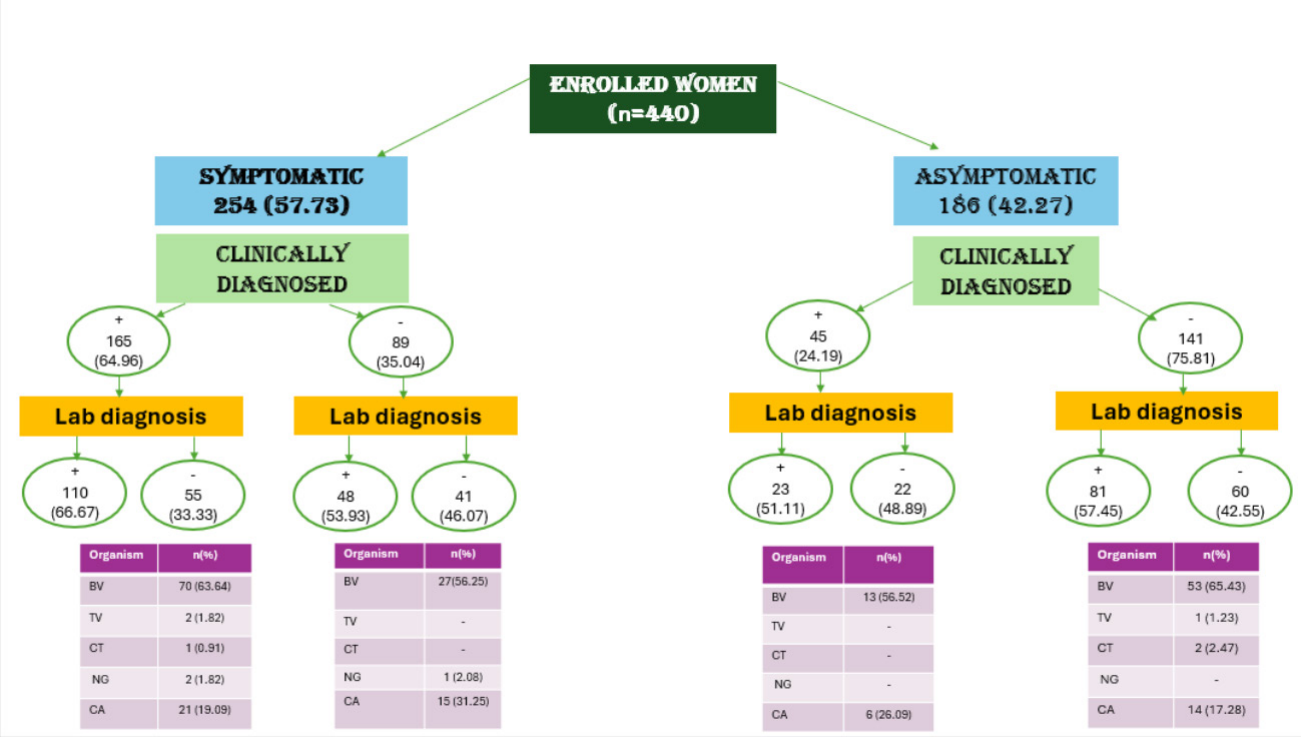
Overview of the prevalence of signs, symptoms and laboratory diagnosis among enrolled women in the study . Among the 440 women enrolled in the study, 254 presented with symptoms and 186 were asymptomatic. Of the symptomatic group, 62.2% had a laboratory-confirmed infection, while 46% tested negative. Among asymptomatic women, 56% had a positive laboratory-confirmed result and 44% tested negative.

254 women presented with symptoms while 186 had no symptoms. 62.2% of symptomatic women had a lab confirmed positive organism while 46% tested negative. Of the 186 asymptomatic women, 56% had a positive lab confirmed report while 44% tested negative. BV was the most prevalent—37%, followed by candida 12%, CT, NG and TV were <1%. BV with CA was the most common combined infection. CT and TV were coinfections with BV. There was one standalone NG infection (figure 1, table 2).

**Table 2:** Prevalence of *Neisseria gonorrhoeae* (NG), *Chlamydia trachomatis* (CT), *Trichomonas vaginalis* (TV), *Bacterial vaginosis* (BV) and *Candida albicans* (CA) among women (n=440)

Prevalence of causative organisms among the women	Prevalence n (%)	95% CI
a. At least one organism		
BV	163 (37.05)	32.52 to 41.75
CA	56 (12.73)	9.76 to 16.21
CT	3 (0.68)	0.14 to 1.98
NG	3 (0.68)	0.14 to 1.98
TV	3 (0.68)	0.14 to 1.98
b. Combination of organisms		
BV and candidiasis	43 (9.77)	7.16 to 12.94
BV and CT	2 (0.45)	0.05 to 1.63
BV and TV	2 (0.45)	0.05 to 1.63
BV and NG	2 (0.45)	0.05 to 1.63
CT and TV	1 (0.23)	0.00 to 1.26

Syndromic diagnosis showed a sensitivity of 60% with 46% specificity, 62% PPV and 44% NPV compared with laboratory confirmed gold standards (table 3). Clinical examination in symptomatic women increased the sensitivity to 66% and PPV to 69% while in asymptomatic women the specificity increased to 73% and PPV was 51%. The NPV for all was between 42% and 44%. Positive likelihood ratios for syndromic and clinical examination in symptomatic cases were both >1 indicating the positive correlation and possibility of presence of RTI/STI, while they were <1 for asymptomatic women (additionally, sensitivity and specificity of vaginitis and cervicitis are mentioned in online supplemental table 2.

**Table 3:** Sensitivity, specificity and predictive values of syndromic approach and clinical diagnosis compared with laboratory-confirmed cases among symptomatic and asymptomatic women

Diagnosis approach	Sensitivity (95% CI)	Specificity (95% CI)	PPV (95% CI)	NPV (95% CI)	Likelihood ratio (+) (95% CI)	Likelihood ratio (−) (95% CI)
Syndromic approach validity vs laboratory diagnosis)	60.31 (54.10 to 66.27)	46.07 (38.58 to 53.68)	62.20 (58.19 to 66.06)	44.09 (38.80 to 49.51)	1.12 (0.95 to 1.32)	0.86 (0.69 to 1.07)
Signs assessment among the symptomatic women vs laboratory diagnosis	66.46 (58.68 to 73.64)	45.56 (35.02 to 56.40)	68.99 (64.14 to 73.45)	42.71 (35.30 to 50.46)	1.22 (0.98 to 1.52)	0.74 (0.54 to 1.01)
Signs assessments among the asymptomatic women vs laboratory diagnosis	22.12 (14.57 to 31.31)	73.17 (62.24 to 82.36)	51.11 (38.62 to 63.47)	42.55 (38.55 to 46.66)	0.82 (0.50 to 1.37)	1.06 (0.90 to 1.26)

Sensitivity=A/(A+C); specificity=D/(B+D); PPV=A/A+B; NPV=C/C+D; positive likelihood ratio=sensitivity/(100− specificity)=sensitivity/(100−specificity); negative likelihood ratio=(100−sensitivity)/specificity.

A: true positive; B: false positive; C: false negative; D: true negative.

NPV, negative predictive value; PPV, positive predictive value.

The only statistically significant association was age, with a 1-year increase in the age of women, there is an 8% reduction in the odds of having a lab-confirmed STI/RTI (OR=0.92) (table 4).

**Table 4:** Multivariable analyses showing the association between demographic, behavioural and sexual factors associated with laboratory-confirmed RTI/STI

Variables	Laboratory-confirmed RTI/STI	
	Adjusted OR (95% CI)	P value
Sociodemographic factors		
Age of the women	0.92 (0.85 to 0.99)	0.03
Women’s years of schooling	1.00 (0.95 to 1.05)	0.98
Occupation of the women	0.96 (0.43 to 2.14)	0.93
Age of the husband	1.03 (0.97 to 1.10)	0.31
Wealth quintile		
Poorest	Reference	
Very poor	1.02 (0.54 to 1.90)	0.96
Poor	1.37 (0.70 to 2.64)	0.35
Less poor	1.00 (0.52 to 1.90)	0.99
Least poor	1.15 (0.59 to 2.25)	0.69
Religion of the head of the household		
Hindu	Reference	
Others	1.37 (0.77 to 2.44)	0.29
Caste		
None of them	Reference	
Other backward classes	1.27 (0.79 to 2.04)	0.32
Scheduled caste	0.74 (0.43 to 1.25)	0.26
Scheduled tribe	0.73 (0.14 to 3.92)	0.39
Family structure		
Nuclear	Reference	
Extended	1.04 (0.66 to 1.62)	0.87
Behavioural characteristics		
Condom used during intercourse	0.72 (0.46 to 1.12)	0.15
Women perceived that their sexual partner had an STI in the past 3 months	2.07 (0.88 to 4.85)	0.09
Intercourse with a sexual partner after returning from travel	0.67 (0.24 to 1.87)	0.46

RTI/STI, reproductive tract infection/sexually transmitted infection.

## Discussion

In our study of 440 women, BV emerged as the most prevalent laboratory confirmed infection followed by Candida. Overall, 60% of women tested positive, including over half of asymptomatic participants, highlighting the hidden burden of infection. A syndromic approach had 60% sensitivity and 46% specificity. Younger participants had significantly higher odds of having an STI, consistent with previous studies showing increased risk among younger age groups.

Vaginal discharge symptom complex was most frequent, followed by itching and lower abdominal pain (online supplemental figure 1). Vaginal infections were often symptomatic, while cervical infections were mostly asymptomatic (online supplemental table 1).

BV followed by candida was also reported as the predominant cause of vaginal discharge in an aetiological surveillance done in South Africa. Although a significant proportion had coinfection with an STI.^5^ Another study involving 200 symptomatic women in an STI clinic in Zimbabwe showed predominance of BV (24.7%) and yeast infection (20.7%).^24^ This is similar to our findings and also highlights the fact that a dysbiotic state like BV is a risk factor for acquisition of STIs. A similar study of 237 women from rural areas and urban slums in Delhi reported lab confirmed BV prevalence at 32.8%.^25^ BV has been consistently reported as the most prevalent infection in studies done at STI clinics in India.^25 26^ Positive lab-confirmed reports were 59.5% in our study including women with and without symptoms compared with a similar study done by Ray *et al* who reported that an aetiological diagnosis could only be established in 32.2% of women, 65% of whom had self-reported symptoms.^11^

A South African study found a high and frequently asymptomatic burden of RTIs in youth with poor sensitivity (8%–20%) of syndromic management compared with the laboratory tests.^27^ Sensitivity and specificity of the syndromic approach in our study were 60% and 46%, respectively, and clinical examination in symptomatic women improved the sensitivity to 66%, PPV from 62% to 68%, while there was no change in specificity with a similar NPV. In a similar trial from a primary health centre in Delhi by Roochika *et al,* the sensitivity improved from 54% to 68% on clinical examination but PPV decreased slightly from 53% to 50%.^28^ Higher sensitivity and PPV are desirable to minimise possibility of overtreatment and development of antimicrobial resistance.

Our study brings a few important issues to ponder. BV is a predominant RTI lending credence to the evidence that the dysbiotic state of the vaginal microbiome is common, can be asymptomatic and there is fairly good evidence about the fact that BV renders women susceptible to more serious infection like HIV and HPV.^29 30^ Untreated BV can potentially be responsible for many adverse reproductive outcomes and we need effective measures to treat BV. Evidence of sexual transmission of BV seems probable, which explains its frequent occurrence.^7 31^ Treatment of the male partner may help, but we need more evidence. A meta-analysis of RCTs by Chen *et al* concluded that probiotics may have a role in the treatment of BV, though it needs more research.^32^

In the current scenario of the STI clinics in India, especially in resource-poor settings, a syndromic approach still remains the best strategy as it ensures immediate treatment and prevents the further spread at the first contact. However, as evident from our study, this approach has poor sensitivity and specificity; therefore, laboratory testing with point-of-care tests would be desirable to make treatment more precise and effective, prevent overtreatment and the possibility of development of antimicrobial resistance. Presently, there are no guidelines for treatment of asymptomatic women, and as evidenced from our study, BV was the most common infection detected. This has a public health implication as BV can render the women susceptible to acquisition of serious STIs, reproductive health morbidity, lead to unavoidable pregnancy losses and preterm birth. An implication, hence our suggestion is to have a system of targeted high-risk screening initiative for all women of reproductive age.

A key strength of this study was the inclusion of asymptomatic women in the study, which enabled a more accurate measure of the burden of RTI/STI in the population and the use of gold standard, NAAT for aetiologic diagnosis. While the collection of samples, their transport, processing and maintaining quality conditions was a challenge, it was mitigated by stringent quality checks. An important limiting factor was that the participants in this study were limited to a prespecified cohort of women sensitised about their health status; hence, a generalisability of the findings should be interpreted with care.

To conclude, BV was the predominant infection (37% followed by candidiasis, CT, NG and TV, which were <1% in this population. The poor diagnostic accuracy of the syndromic approach (sensitivity 60%, specificity 46%) highlights the need for enhanced diagnostic strategies. The high prevalence among asymptomatic and younger women warrants greater awareness and targeted intervention to reduce the RTI/STI burden and its consequences.

## Supplementary material

10.1136/bmjph-2024-001791 online supplemental file 1

## Data Availability

Data are available on reasonable request.

## References

[1] WHO fact sheet on sti. https://www.who.int/news-room/fact-sheets/detail/sexually-transmitted-infections-(stis).

[2] Institute of medicine committee on pcost, diseases (1997). The Hidden Epidemic: Confronting Sexually Transmitted Diseases: Summary.

[3] Department of AIDS Control (2014). Ministry of health and family welfare government of India. prevention, management and control of reproductive tract infections and sexually transmitted infections.

[4] Workowski KA, Bolan GA, Centers for Disease Control and Prevention (2015). Sexually transmitted diseases treatment guidelines, 2015. *MMWR Recomm Rep*.

[5] Lewis FMT, Bernstein KT, Aral SO (2017). Vaginal Microbiome and Its Relationship to Behavior, Sexual Health, and Sexually Transmitted Diseases. Obstet Gynecol.

[6] Hay P (2017). Bacterial vaginosis. F1000Res.

[7] WHO Key facts on bacterial vaginosis. https://www.who.int/news-room/fact-sheets/detail/bacterial-vaginosis.

[8] Desai VK, Kosambiya JK, Thakor HG (2003). Prevalence of sexually transmitted infections and performance of STI syndromes against aetiological diagnosis, in female sex workers of red light area in Surat, India. Sex Transm Infect.

[9] Department of Reproductive Health and Research, World Health Organization (2012). Strategies and laboratory methods for strengthening surveillance of sexually transmitted infection. https://www.who.int/reproductivehealth/publications/rtis/9789241504478/en.

[10] Taneja S, Chowdhury R, Dhabhai N (2020). Impact of an integrated nutrition, health, water sanitation and hygiene, psychosocial care and support intervention package delivered during the pre- and peri-conception period and/or during pregnancy and early childhood on linear growth of infants in the first two years of life, birth outcomes and nutritional status of mothers: study protocol of a factorial, individually randomized controlled trial in India. Trials.

[11] Ray K, Muralidhar S, Bala M (2009). Comparative study of syndromic and etiological diagnosis of reproductive tract infections/sexually transmitted infections in women in Delhi. Int J Infect Dis.

[12] Dhabhai N, Chaudhary R, Wi T (2022). Prevalence of reproductive tract infections including sexually transmitted infections among married women in urban and peri-urban mid to low socioeconomic neighbourhoods of Delhi, North India: an observational study protocol. BMJ Open.

[13] Cox C, McKenna JP, Watt AP (2015). New assay for Gardnerella vaginalis loads correlates with Nugent scores and has potential in the diagnosis of bacterial vaginosis. J Med Microbiol.

[14] World Health Organization (2012). Strategies and laboratory methods for strengthening surveillance of sexually transmitted infection, 2012. https://iris.who.int/bitstream/handle/10665/75729/9789241504478_eng.pdf.

[15] Jacobsson S, Boiko I, Golparian D (2018). WHO laboratory validation of XpertCT/NG and Xpert TV on the GeneXpert system verifies high performances. APMIS.

[16] Zeptometre, women’s health &STI controls & panels for molecular diagnostic QC. https://www.zeptometrix.com/products/tvaginalis-verification-panel-17-x-07-ml.

[17] https://www.zeptometrix.com/products/ct-ng-panel-17-x-12-ml.

[18] Zepto-metre NATtrol whole orgaism controls. https://www.zeptometrix.com.

[19] CBNAAT gene xpert platform is an automated in vitro C and differentiation of dna from CT, NG. https://www.cepheid.com/en/systems/GeneXpert-Family-of-Systems/GeneXpert-System.

[20] The genexpert CT/NG cepheid IVD, and genexpert used to detect CT, NG, and TV. https://www.cepheid.com/en/tests/Sexual-Health/Xpert-CT-NG.

[21] National guidelines on prevention (2006). Management and control of reproductive tract infections including sexually transmitted infections. http://naco.gov.in/sites/default/files/National_Guidelines_on_PMC_of_RTI_Including_STI%201.pdf.

[22] Bursac Z, Gauss CH, Williams DK (2008). Purposeful selection of variables in logistic regression. Source Code Biol Med.

[23] Hosmer DW, Lemeshow S, Sturdivant RX (2013). Applied Logistic Regression.

[24] Chirenje ZM, Dhibi N, Handsfield HH (2018). The Etiology of Vaginal Discharge Syndrome in Zimbabwe: Results from the Zimbabwe STI Etiology Study. Sex Transm Dis.

[25] Bhalla P, Chawla R, Garg S (2007). Prevalence of bacterial vaginosis among women in Delhi, India. Indian J Med Res.

[26] Nair RV, Preethi R, Vijayalekshmi M (2019). Prevalence of bacterial vaginosis among reproductive age group women in a tertiary care centre. Int J Reprod Contracept Obstet Gynecol.

[27] Kaida A, Dietrich JJ, Laher F (2018). A high burden of asymptomatic genital tract infections undermines the syndromic management approach among adolescents and young adults in South Africa: implications for HIV prevention efforts. BMC Infect Dis.

[28] Ranjan R, Sharma AK, Mehta G (2003). Evaluation of WHO Diagnostic Algorithm for Reproductive Tract Infections among Married Women. Indian J Community Med.

[29] Atashili J, Poole C, Ndumbe PM (2008). Bacterial vaginosis and HIV acquisition: a meta-analysis of published studies. AIDS.

[30] Martins BCT, Guimarães RA, Alves RRF (2023). Bacterial vaginosis and cervical human papillomavirus infection in young and adult women: a systematic review and meta-analysis. Rev Saude Publica.

[31] Philippe H G (2025). Bacterial Vaginosis. Drugs & Diseases.

[32] Chen R, Li R, Qing W (2022). Probiotics are a good choice for the treatment of bacterial vaginosis: a meta-analysis of randomized controlled trial. Reprod Health.

