# Supplementary material for: Prevalence of reproductive tract and sexually transmitted infections among symptomatic and asymptomatic women, validity of syndromic management, in urban and periurban low to mid socioeconomic neighbourhoods of North Delhi: an observational study
Source: BMJ Public Health. 2025 Oct 27;3(2):e001791. doi: 10.1136/bmjph-2024-001791 (PMC12574350; doi:10.1136/bmjph-2024-001791)
Supplement: online supplemental file 1 [file bmjph-3-2-s001.docx]

**Supplemental material**

**Definitions**

**Sensitivity** Proportion of women having syndromic diagnosis of infection among those who had a laboratory confirmed infection.

**Specificity** Proportion of women having syndromic diagnosis of no infections among those who had no laboratory confirmed infection.

**Positive predictive value (PPV)** Proportion of women with a laboratory diagnosed infection among those had syndromic diagnosis of infections.

**Negative predictive value (NPV)** Proportion of women with no laboratory confirmed infections among those did not have syndromic diagnosis of infections .

**Positive likelihood ratio**- sensitivity/ (1-specificity)

**Negative likelihood ratio** (1-sensitivity)/specificity.

**Figure 1**

**Table 1. Clinical diagnosis among symptomatic and asymptomatic women**

| **Clinical Diagnosis** | **Symptomatic women (N=254)** | **Asymptomatic women (N=186)** |
| --- | --- | --- |
|  | **n (%)** | **n (%)** |
| Vaginitis | **46 (28.05)** | 9(20.00) |
| Cervicitis and vaginitis | 31(18.90) | 6(13.33) |
| Cervicitis with Pelvic inflammatory disease | 10(6.10) | 1(2.22) |
| Cervicitis | 17 (10.37) | **21 (46.67)** |
| Pelvic inflammatory disease | 14 (8.54) | 5 (11.11) |
| Vaginitis and pelvic inflammatory disease. | 20 (12.20) | - |
| Vulvitis | 5(3.05) | 1(2.22) |

**Table 2. Sensitivity, specificity, predictive values and likelihood ratios of syndromic approach and clinical diagnosis compared to laboratory-confirmed cases of vaginitis and cervicitis among symptomatic women**

| **Parameters** | **Vaginitis** | **Cervicitis** |
| --- | --- | --- |
| Sensitivity | 0.83 | 0.91 |
| Specificity | 0.38 | 0.15 |
| Positive predictive value | 0.65 | 0.65 |
| Negative predictive value | 0.33 | 0.41 |
| Positive likelihood ratio | 1.34 | 1.07 |
| Negative likelihood ratio | 0.45 | 0.60 |
